# Interactions in the aetiology, presentation and management of synchronous and metachronous adenocarcinoma of the prostate and rectum

**DOI:** 10.1308/003588412X13373405384611

**Published:** 2012-10

**Authors:** GF Nash, KJ Turner, T Hickish, J Smith, M Chand, BJ Moran

**Affiliations:** ^1^Poole Hospital NHS Foundation Trust,UK; ^2^Bournemouth University,UK; ^3^Hampshire Hospitals NHS Foundation Trust,UK

**Keywords:** Pelvic cancer, Adenocarcinoma, Prostate, Rectum

## Abstract

Adenocarcinoma of the prostate and rectum are common male pelvic cancers and may present synchronously or metachronously and, due to their anatomic proximity. The treatment of rectal or prostate cancer (in particular surgery and/or radiotherapy) may alter the presentation, incidence and management should a metachronous tumour develop. This review focuses on the interaction between prostatic and rectal cancer diagnosis and management. We have restricted the scope of this large topic to general considerations, management of rectal cancer after prostate cancer treatment and vice versa, management of synchronous disease and cancer follow-up issues.

Adenocarcinoma of the prostate and rectum are common male pelvic cancers and both increase in incidence with age. The incidence of prostatic cancer is higher than rectal cancer but cancers of both organs can present synchronously or metachronously and due to their anatomic proximity (Fig 1), an advanced cancer of either may directly invade the other. As both prostatic and rectal cancer are more common with age, synchronous tumours may be incidental although there is evidence to suggest that aetiology may be linked.^1^ Certainly, environmental factors such as dietary fat seem to promote both cancers. A further confounding feature is that treatment of rectal or prostate cancer (in particular surgery and/or radiotherapy) may alter the presentation, incidence and management should a metachronous tumour develop. Overall, both prostate and rectal cancer diagnoses are increasing although this may be partly attributable to awareness and better diagnostic methods.^2^

**Figure 1:**
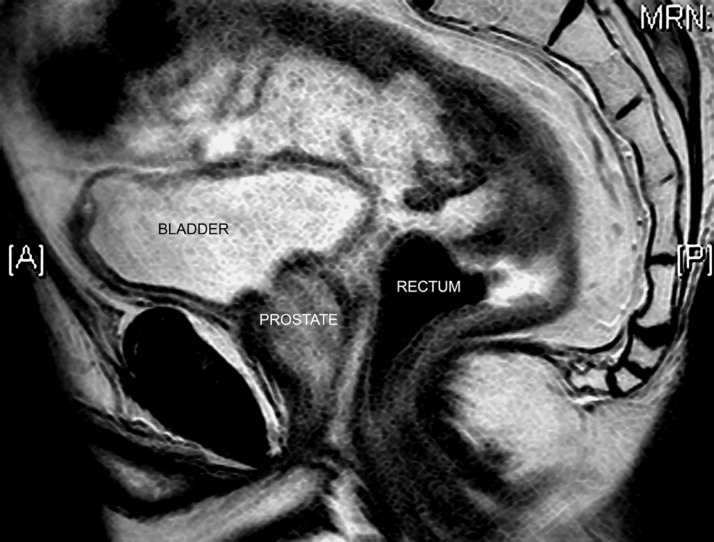
Midline sagittal pelvic magnetic resonance imaging demonstrating the proximity of the rectum and prostate

Despite their distinct sites in two separate body systems, the symptoms, signs and investigations of both conditions may be confusing and sometimes difficult to disentangle. Additionally, in patients diagnosed with synchronous rectal and prostate malignancies or metachronous presentation, management options are often compromised by the initial cancer treatment. This review focuses on the interaction between prostatic and rectal cancer diagnosis and management. We have restricted the scope of this large topic to general considerations, management of rectal cancer after prostate cancer treatment and vice versa, management of synchronous disease and cancer follow-up issues.

## Methods

A literature search was performed using PubMed, Embase™, Ovid® and Google search engines. The search included all articles up to and including November 2011 and was limited to papers in the English language and those translated into English only. A combination of the following search headings was used: ‘rectal cancer’, ‘prostate cancer’, ‘radiotherapy’, ‘radiation’, ‘surgery’, ‘radiation proctitis’, ‘CEA’ [carcinoembryonic antigen], ‘PSA’ [prostate specific antigen] and ‘screening’. Technical reports, editorials and studies of patients aged under 16 years were excluded. There was manual cross-referencing of the yield. Any further articles identified were assessed against the inclusion/exclusion criteria before undergoing more detailed assessment.

## General considerations concerning prostate and rectal cancer

In recent years, screening programmes have been developed for both diseases as both prostate and rectal cancer may be detected prior to the onset of symptoms. Nevertheless, both diseases may also present late with advanced cancer metastases when the patient complains of weight loss and general malaise. In men of middle age onwards the symptoms of prostate and rectal cancer may overlap and mislead both the patient and medical carers. It is well established that many cancers of the prostate and the lower part of the rectum may be palpable by digital rectal examination (DRE). However, particularly to the inexperienced, DRE alone may not diagnose the correct cancer clearly and, even for experienced clinicians, DRE alone is not sensitive enough to diagnose prostate cancer reliably.

An exophytic endoluminal mass is more commonly associated with rectal cancer whereas prostatic cancer growth is typically extraluminal. Infiltration of the rectum by prostate cancer is found in approximately 4% of cases and this usually indicates advanced disease with a poor prognosis.^3^ Less commonly, an annular stricture of the rectum may be caused by more extensive prostatic infiltration encircling the rectum,^4^ which may present with faecal urgency.

Tumour markers, specifically CEA and PSA, can be helpful both in diagnosis and during the follow-up period. CEA may rise during chemotherapy or radiotherapy for prostate cancer, possibly by systemic CEA release as a result of tumour cell death. Similarly, PSA may increase initially following rectal cancer radiotherapy but this rise often diminishes in the long term.^5^

Nevertheless, it should be remembered that both CEA and PSA may rise in many benign diseases. Further discussion of CEA is beyond the scope of this review. Persistently abnormal levels of tumour markers should, however, prompt further investigation even if such benign conditions associated with elevated tumour markers are present.

## Management of rectal cancer following prior prostate cancer treatment

### Incidence and presentation

As the rectum is immediately posterior to the prostate, the anterior wall of the rectum in particular receives a dose of external beam radiotherapy (EBRT) similar to the dose received by the prostate.^6^ Certainly, EBRT for prostate cancer patients delivers a dose of radiation to the pelvic rectum much higher than the dose received by the colon. Therefore, one might hypothesise that EBRT, with its local effect, might increase rectal cancer incidence compared with colon cancer and, overall, approximately a third of colorectal cancers are found in the rectum.^2^ There does seem to be some evidence of variance from this assumption with some studies showing no increase in the post-radiation rectal cancer rate. Indeed, one study showed that colonic rather than rectal cancer incidence was increased.^7^

A number of studies have concluded that rectal cancer is more common following radiotherapy for prostate cancer^8–11^ and the majority of oncologists believe there is a correlation. The lifetime risk of rectal cancer is approximately 2%^2^ and most studies estimate an additional 1% risk of developing rectal cancer following radiotherapy. A study from 2006 found no association between rectal cancer and radiotherapy.^12^ The authors suggested factors such as selection bias, patient age, study size and statistical analysis to possibly explain the differing results of other studies.^13^

Undoubtedly, radiotherapy administration techniques have become more precise over recent years such that collateral damage is likely to be reduced compared with the past. The conflicting results of research studying the relationship between radiotherapy and rectal cancer incidence may be explained partially by the different doses and modalities of radiotherapy delivery as, for example, it is suggested that radioactive implants may have no added risk of secondary cancers.^11^ As many of the effects of radiotherapy may manifest decades later, long-term follow-up is needed and data set completeness is likely to be suboptimal.

**Table 1:** National Institute for Health and Clinical Excellence risk stratification criteria for men with localised prostate cancer^25^

	PsA (ng/ml)		Gleason score		Clinical stage
Low risk	<10	and	≤6	and	T1–T2a
Intermediate risk	10–20	or	7	or	T2b–T2c
High risk	>20	or	8–10	or	T3–T4

National Institute for Health and Clinical Excellence. *CG58 Prostate Cancer*. London: NICE; 2008 (available from www.nice.org/CG58). Reproduced with permission.

Although the maximum tolerable pelvic radiation dose is not understood clearly,^14^ it may be technique and patient dependent. Several studies have investigated the radiation dose-volume effects in radiation induced rectal injury, particularly after treatment of prostate cancer in men and gynaecological cancer in women. It has been well established that patients receiving more than 60Gy consistently develop rectal bleeding,^15^ which may occur episodically over a period of decades following radiotherapy.

### Rectal cancer assessment and therapy following prostate irradiation

Magnetic resonance imaging (MRI) is carried out routinely to assess the circumferential margin of rectal cancer and it may identify extension into the prostate.^16^ When the resection margin is involved or threatened by extent of the rectal cancer through the wall of the rectum, pre-operative radiotherapy would usually be considered. In addition, surgery for rectal cancer becomes more challenging following radiotherapy for prostate cancer as the planes surrounding the rectum become more friable and blood loss is increased.^17^

Following pelvic radiotherapy, probably as a result of ischaemia,^18^ the healing potential of the rectum is reduced and the morbidity following anterior resection can be increased tenfold.^19^ A particular risk is anastomotic leak, which carries a high mortality rate and may also be associated with an increased risk of local recurrence.^20^ For this reason, a temporary stoma to defunction a low anterior resection following prior radiotherapy is recommended. However, although a temporary loop stoma may reduce the adverse consequences of a leak, it appears to not change the leak rate significantly per se.^21^

The standard treatment for advanced low rectal cancer (staged pre-operatively and predicted to be advanced T3 or T4 disease) in many institutions is currently pre-operative chemoradiation followed by total mesorectal excision although this strategy has double the major complication rate compared with surgery alone.^22^ If patients have had prior pelvic radiation, further radiotherapy is contraindicated and may not be used for its known downstaging and downsizing effect on rectal cancer. The omission of radiotherapy in advanced cases may lead to an increased post-operative local recurrence rate. Nevertheless, there is some evidence that neoadjuvant chemotherapy may have a role in this setting.^23^

## Management of prostate cancer following prior rectal cancer surgery

The management of prostate cancer after prior rectal cancer surgery is influenced by the nature of the previous surgery, whether neoadjuvant or post-operative chemoradiotherapy has been used, the prognosis of the patient with regard to the rectal cancer and the patient’s general fitness. Pre-existing functional deficits, in terms of bowel and genitourinary function, also need to be taken into account when planning intervention for prostate cancer. There are a number of relevant issues regarding the diagnosis and treatment options for prostate cancer after prior rectal cancer treatment as outlined below.

### Diagnosis of prostate cancer

The population of patients who have had radical treatment for rectal cancer are of a similar age and demographics to the age group considered for PSA screening. Currently, there is no consensus in the UK on the use of PSA screening for prostate cancer detection. There is general agreement that asymptomatic patients found to have a raised PSA should undergo a DRE. However, if a patient has had an abdominoperineal excision (APE), prostate palpation is impossible. In addition, there are difficulties with PSA assays in combination with DRE in the distinction of aggressive versus indolent prostate cancer although it has been reported that the level of the baseline PSA correlates with the development of worse prognosis prostate cancer.^24^

Guidance from the UK National Institute for Health and Clinical Excellence (NICE) suggests that men should not be offered a biopsy on the basis of elevated PSA alone.^25^ Other factors should be considered such as DRE findings, estimate of prostate size and PSA level, PSA density, previous negative biopsies, family history, age, race, co-morbidity and prognosis from other cancers. Following histological diagnosis, NICE advocates using the PSA level as well as Gleason score and clinical stage to stratify men into three risk categories: low, intermediate and high risk.^25^

There is less controversy regarding treatment of men with intermediate or high risk disease. The group with low risk, localised prostate cancer constitutes the area of maximum controversy in terms of overdiagnosis and overtreatment. NICE recommendations, as a key priority, are that all men with low risk, localised disease be offered active surveillance.^25^

Patients with raised PSA after radical rectal cancer treatment should have DRE performed unless they have had an APE where DRE is impossible. If the PSA, history and clinical findings are suspicious, then a biopsy can be planned. If accessible transanally, transrectal ultrasonography (TRUS) can visualise the prostate and facilitate biopsy. TRUS biopsies of the prostate may also be performed transperineally if rectal mucosa trauma is undesirable (eg if there is carpet polyp in the rectum or a recent rectal anastomosis). If TRUS is not possible (for example, in patients post-APE), a prostate biopsy may be performed guided by other imaging modalities (ultrasonography, computed tomography [CT] or MRI).

Following histological diagnosis, the need and role for further imaging should be considered in a multidisciplinary meeting. NICE recommendations are for ‘pelvic imaging’ in the form of MRI or CT (where MRI is contraindicated) only in the high risk prostate cancer population (PSA >20ng/ml, Gleason score 8–10 or clinical T3/T4 disease).^25^ A change in symptoms in men undergoing active surveillance for prostate cancer may prompt restaging investigations. If there is a rectal carcinoma in situ and a synchronous prostate cancer, the possibility of implanting rectal adenocarcinoma may be avoided by a transperineal biopsy of the prostate. However, transperineal template biopsies may indicate more significant disease, leading to radical prostate cancer treatment rather than continued surveillance.^26^

MRI has superior soft tissue contrast, spatial resolution and interplanar distinction to CT and helps to stage the prostate cancer as well as to assess tissue planes between the prostate and rectum. There is ongoing work in progress to determine whether MRI will, in due course, be sufficiently accurate in staging prostate cancer and allow some patients to avoid prostatic biopsy with its attendant morbidity.

Following rectal cancer surgery, the presence of infection, haemorrhage, atrophy or post-focal treatment changes can have similar signal appearances and can result in difficulties in MRI interpretation. In the future, other MRI-based techniques including diffusion weighted MRI, MRI spectroscopy and dynamic contrast enhanced MRI may become more specific imaging modalities in this scenario.

## Treatment options for prostate cancer following prior rectal cancer treatment

Provided the prognosis for rectal cancer outcome is satisfactory, the decision making process focuses on the patient’s prostate cancer. Disease should be stratified by PSA, clinical stage, Gleason score, volume of disease on biopsy and radiological stage. Options include active surveillance, intervention with curative intent, immediate androgen deprivation therapy (ADT) and deferred ADT.

As stated previously, NICE recommends that active surveillance should be offered to all men with low risk disease: PSA <10ng/ml, Gleason score 6, clinical stage T1c/T2a.^25^ Active surveillance in men with localised disease, who would be suitable for radical treatment, aims to avoid treatment and possible morbidity of overtreatment. During the period of observation, if there is an increase in Gleason score, disease volume, a change in DRE findings or an increase in PSA, the patient management may change from surveillance to radical intervention with curative intent.

### Radical treatment

The nature of previous rectal cancer treatment will dictate the options for prostate cancer treatment. Radical options for the treatment of prostate cancer include radical prostatectomy, external beam radiotherapy and brachytherapy. Patients who have had T1/2 rectal cancer treatment may have had primary surgery with no neoadjuvant or adjuvant treatment and are theoretically suitable for all options. However, patients who have undergone prior treatment that has involved neoadjuvant chemoradiotherapy would have had a regime that likely included 45–50Gy radiotherapy to the pelvis^27^ and therefore cannot have high dose energy prostate cancer treatments, leaving radical prostatectomy as the only radical option.

Nevertheless, there are methods in development, including balloon devices, that may both stabilise the position of the prostate and separate it from the rectum to reduce the side effects of prostate irradiation on the rectum and vice versa. In addition, brachytherapy is impossible following APE as most patients have had radiotherapy as part of their rectal cancer treatment and no access to allow siting of the radioactive seeds.

In a similar fashion to the increased risks of complications in rectal cancer surgery following radiotherapy, the increased morbidity of prostatectomy after previous pelvic radiotherapy includes incontinence/lower urinary tract symptoms, erectile dysfunction and rectourethral fistula (RUF). Recent reports suggest that currently more than 50% of patients with RUF have followed EBRT, brachytherapy or a combination of these.^28^ The reported incidence of brachytherapy induced RUF has been reported to be up 5%.^29^

There are no large studies on of the outcomes of radical prostatectomy following pelvic radiotherapy for rectal cancer. However, various surgical strategies have been employed historically to reduce the risk of RUF formation with omental interposition being one of the more common techniques. In terms of the surgical approach, during a retropubic radical prostatectomy, the radiation distorted posterior plane is at increased risk of rectal injury, which may be best managed by primary closure prior to omental interposition and a defunctioning stoma. Bowel preparation and consent for a stoma, if needed, is recommended.

## Management of synchronous prostate and rectal cancer

**Figure 2:**
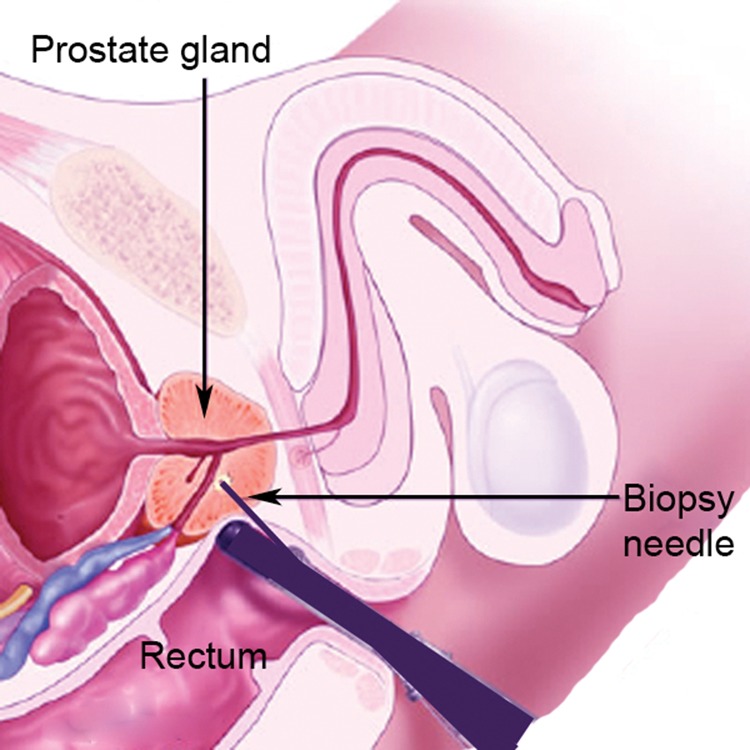
Diagram of midline structures in the pelvis demonstrating transanal biopsy of the prostate

Due to the anatomical relationship between the prostate and rectum, erroneous diagnosis of prostate rather than rectal neoplasia and vice versa at ultrasonography or biopsy must be borne in mind. Ultrasonography of the anorectum is used increasingly in early rectal cancer to determine T staging and may diagnose prostate cancer coincidentally.^30,31^ Transanal biopsy (Fig 2) is the simplest method for gaining a definitive histological diagnosis for both rectal and prostate neoplasia. The clinician should be aware that prostatic biopsy may produce symptoms for a few weeks of bleeding, tenesmus and rectal discomfort, which may mimic rectal neoplasia.

The finding of rectal tissue following a biopsy of the prostate is common as the needle must transverse the rectal wall and Denonvilliers’ fascia. Nevertheless, distorted rectal tissue may occasionally display some features consistent with prostate adenocarcinoma. It is important that biopsies are assessed by an experienced pathologist, who may identify some of the misleading features, thereby avoiding misdiagnosis. In some cases, the diagnosis of coexisting prostate cancer following surgical treatment for rectal cancer may only become apparent after the lymph nodes are histologically examined following a mesorectal excision.^32^

Synchronous disease is rarer than metachronous rectal and prostate cancer and therefore makes management decisions on synchronous cancer even more difficult. However, it is important to make sure that there are indeed two separate cancers rather than one presenting atypically. As there are several cases described of inappropriate rectal surgery for an infiltrating prostatic cancer,^3^ it is important to verify synchronous rectal and prostatic cancers histologically. In synchronous disease, accurate CT and MRI staging is crucial to evaluate the extent of both malignancies to reduce the chance of unnecessary radical surgery.

There are a number of small case series but no large reported series of synchronous cancer treatment outcomes. Radiotherapy has been described as an effective treatment for synchronous cancers^33^ and may be more appropriate in less fit patients, as may hormonal therapy in combination with radiation. Although successful combined radical prostatectomy and rectal cancer surgery is possible following pelvic radiation,^34^ the surgeon is wise to approach such cases with caution and to consent the patient carefully regarding the major morbidity of bleeding, sepsis, impotence and incontinence. Urinary incontinence develops in almost a third of patients after rectal cancer surgery, and combined urinary and faecal incontinence occurs in 14% of patients with normal pre-operative function.^35,36^

The effect of rectal cancer surgery is often to reduce the rectal capacity, which, combined with radiotherapy, commonly reduces the ability to defer defaecation.^37^ Indeed, the incidence of faecal incontinence following pelvic radiotherapy alone has been reported to be as high as 58%.^38^

## Longer term effects of radiotherapy and patient follow-up

### Misdiagnosis of rectal cancer due to radiation proctitis

Two of the most common symptoms of colorectal cancer are rectal bleeding and an increased bowel frequency. Similarly, diarrhoea and bleeding are the most common symptoms from radiation proctitis and occur to some degree in the majority of cases.^39^ Other typical post-radiation symptoms include tenesmus, urgency and pelvic pain. Histopathological findings in radiation proctitis are typically obliterating arteritis with fibrosis. However, tissue biopsy may be inconclusive and the diagnosis of late radiation proctitis may require exclusion of rectal cancer.

**Figure 3:**
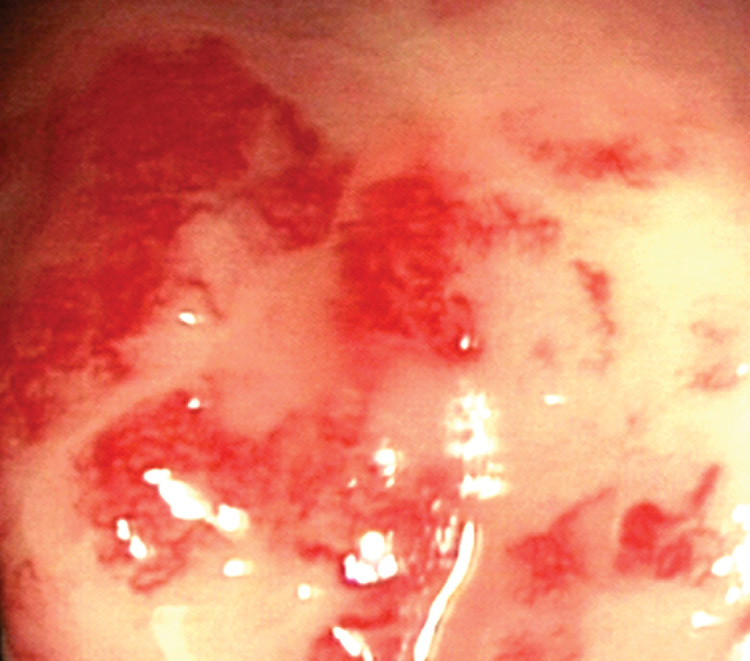
The characteristic appearance of chronic radiation proctitis

Chronic radiation proctitis can follow on from the acute phase or may commence many years later. A large study of patients who had pelvic cancer radiotherapy reported that those suffering acute radiotherapy induced diarrhoea tended to also suffer the late complications.^40^ Although acute toxicity may predict a higher risk of chronic proctitis, this is not necessarily the case.^41^ The natural history of late radiation proctitis for an individual is difficult to determine but severe complications are more likely to be associated with persistent bleeding strictures.^42^ Radiological imaging may suggest radiation damage when a lack of rectal distension is seen due to fibrosis^43^ but as the mucosa is the worst affected site, proctoscopy is a more sensitive investigation showing characteristic telangiectatic lesions often arranged linearly in the anterior part of the rectum (Fig 3).

### Treatment of radiation proctitis

Many treatments have been suggested and used to improve and limit the symptoms of radiation proctitis although there is no strong evidence base for these.^44^ Aminosalicylic acid enemas and oral metronidazole are commonly used as first line treatments. Argon beam coagulation has been reported as effective and safe, and intrarectal 4% formalin instillation seems to be effective but possibly has a higher rate of complications.^45^ If available, the use of hyperbaric oxygen may also be an option for resistant cases.

### Future cancer risk reduction

Radiation induced rectal cancer is probably a consequence of the carcinogenic effects of ionising radiation but may also be related to chronic inflammation. There is accumulating evidence that non-steroidal anti-inflammatory drugs (NSAIDs) reduce the risk of primary colorectal cancer, possibly by their inhibition of prostaglandins.^46^ Prostaglandins may increase the carcinogenic potential of cells through the oxidation of precarcinogens to carcinogens. The antineoplastic effects of NSAIDs may also occur through increased apoptosis, decreased cell growth or an altered immune response to cancer cells. It would appear that although aspirin may reduce the incidence of many cancers, for both prostate and rectal cancer this risk reduction may be an effect observed only after many years on aspirin.^47^ The role of inflammation in the development of prostate cancer needs further research as does the use of anti-inflammatories in this setting, especially as bleeding from radiation proctitis may be exacerbated by these agents.

It is interesting to note that the subsequent incidence of metachronous prostate cancer seems to be reduced by radiation used in the treatment of rectal cancer.^48,49^ This decreased risk of prostate cancer in men who have received radiation therapy for rectal cancer may be related to the curative effect on occult prostate cancer. Dysplasia in the prostate gland is very common in later life and it may be that radiotherapy retards the progression of these dysplastic changes. Overall, the true incidence of second primary tumours in adjacent pelvic organs represents a balance between those radiation induced tumours and the radiation inhibition of spontaneously occurring tumours.^50^

The suggested increased incidence of rectal cancer following pelvic radiotherapy makes this population ideal for regular screening of the rectal mucosa. The timing of proctosigmoidoscopy must be considered carefully and great care must be exercised with any biopsy after brachytherapy.^51^ Worryingly, there is even the possibility that biopsy of areas close to the prostate may extract a radioactive seed. The necessity of rectal biopsy should therefore be considered carefully and if a biopsy is taken, it would seem sensible to avoid the anterior rectum.

NICE guidance suggests that patients presenting with symptoms consistent with radiation induced enteropathy should be fully investigated^25^ (including using flexible sigmoidoscopy) to exclude inflammatory bowel disease or malignancy of the large bowel and to ascertain the nature and extent of the radiation injury. NICE guidance also recommends that men treated with radical radiotherapy for prostate cancer should be offered flexible sigmoidoscopy every five years.^25^ However, radiotherapy may also increase the rate of other cancers such as sarcomas, which are not necessarily mucosal in origin and may be missed by flexible sigmoidoscopy.^52^ After pelvic radiotherapy, even if five-yearly follow-up surveillance of the rectal mucosa is planned, there should be a low threshold for investigation of rectal bleeding to avoid missing interval cancers as it cannot be assumed that the usual polyp-cancer sequence occurs with radiation induced cancers.

The long-term survival probability after five years exceeds 90% for locally staged and curatively treated prostate or rectal cancer.^53^ There is evidence to support longer term follow-up after prostate cancer treatment as cancer specific survival continues to decline up to and beyond 15 years after diagnosis whereas beyond 10 years, survival after treatment of rectal cancer remains constant.^2^

## Conclusions

Prostate and rectal cancers are common in men and a major cause of cancer death. Due to the close anatomical relationship, adenocarcinoma of the rectum and prostate overlap in presenting symptoms, and cancers of both organs may present synchronously or metachronously. Both cancers are detectable by screening methods and are readily amenable to curative treatment, especially when detected at an early stage. There may be an association in aetiology and the treatment of one cancer can impact on the presentation and therapy of the other.

A potential bias towards PSA screening in patients who have had previous cancers such as rectal cancer puts men at risk of overdiagnosis and overtreatment with its accompanying significant morbidity. The importance of accurate disease stage and grade is crucial when making treatment decisions for prostate and rectal cancers occurring either synchronously or metachronously. Thus, for prostate cancer, radical prostatectomy after major treatment for rectal cancer has increased risks both in terms of functional outcomes and, in particular, morbidity such as post-operative RUFs. These issues need to be considered and special measures adopted to reduce risk and complications. It is important to consider rectal cancer as the cause of bleeding following prior pelvic radiation as rectal cancer may be more common after radiation. Furthermore, symptoms may be overlooked by patients and carers and mistakenly attributed to radiation proctitis.

The interaction between rectal and prostate cancer is common, complex and worth keeping in mind as both cancers are amenable to cure whether presenting synchronously or in a metachronous sequence.
